# Causes of death and demographic characteristics of victims of meteorological disasters in Korea from 1990 to 2008

**DOI:** 10.1186/1476-069X-10-82

**Published:** 2011-09-27

**Authors:** Hyung-Nam Myung, Jae-Yeon Jang

**Affiliations:** 1 Department of Preventive Medicine and Public Health, Ajou University School of Medicine, Suwon, Korea

**Keywords:** climate change, disasters, flood, typhoon, death rate, vulnerability, epidemiology

## Abstract

**Background:**

Meteorological disasters are an important component when considering climate change issues that impact morbidity and mortality rates. However, there are few epidemiological studies assessing the causes and characteristics of deaths from meteorological disasters. The present study aimed to analyze the causes of death associated with meteorological disasters in Korea, as well as demographic and geographic vulnerabilities and their changing trends, to establish effective measures for the adaptation to meteorological disasters.

**Methods:**

Deaths associated with meteorological disasters were examined from 2,045 cases in Victim Survey Reports prepared by 16 local governments from 1990 to 2008. Specific causes of death were categorized as drowning, structural collapse, electrocution, lightning, fall, collision, landslide, avalanche, deterioration of disease by disaster, and others. Death rates were analyzed according to the meteorological type, specific causes of death, and demographic and geographic characteristics.

**Results:**

Drowning (60.3%) caused the greatest number of deaths in total, followed by landslide (19.7%) and structural collapse (10.1%). However, the causes of deaths differed between disaster types. The meteorological disaster associated with the greatest number of deaths has changed from flood to typhoon. Factors that raised vulnerability included living in coastal provinces (11.3 times higher than inland metropolitan), male gender (1.9 times higher than female), and older age.

**Conclusions:**

Epidemiological analyses of the causes of death and vulnerability associated with meteorological disasters can provide the necessary information for establishing future adaptation measures against climate change. A more comprehensive system for assessing disaster epidemiology needs to be established.

## Background

Patterns of meteorological elements such as temperature and precipitation have been altered due to climate change [1]. The frequency, intensity, and duration of meteorological disasters have also increased since the 1920s [2,3]. These phenomena are attributed to the accelerated rise in the sea level due to climate changes, increasing sea surface temperatures, more powerful tropical and temperate cyclones, changing characteristics of air pressure and precipitation, and acidification of the oceans [1,4,5]. Meteorological disasters, along with extreme heat, infectious diseases, water- and food-borne illnesses, and air pollution, are important components of climate change that impact morbidity and mortality rates [6-8].

Studies of damages caused by meteorological disasters, however, usually address economic loss and rarely deal with disaster epidemiology regarding victims and vulnerable groups [9]. It is estimated that climate change will worsen the frequency and severity of meteorological disasters in the future [7,10,11]. There are several examples of this trend, involving massive numbers of victims caused by powerful meteorological disasters in recent years, including the Sri Lanka tsunami of 2004 [12] and hurricane Katrina in the US in 2005 [13]. It is also estimated that poorer countries will have more victims as a result of meteorological disasters, because of the huge disease burden [10,14]. Thus, victims of meteorological disasters will emerge as a critical social and public health issue on a global level.

Information regarding specific causes of death due to meteorological disasters is essential to identify vulnerable population groups and establish preventive measures against damages, and to recommend behavioral guidelines for citizens. Thus, it is important to analyze specific causes of death when we study the demographic and regional characteristics of deaths due to meteorological disasters according to countries and regions.

Some studies have analyzed causes of death according to specific types of disasters such as floods, tsunamis, and hurricanes [12,15-17]. However, it is difficult to find research that concretely analyzes causes of death and sociodemographic characteristics over a long time-period beyond the accumulation of the number of victims by meteorological disaster.

A previous study [2] examined trends in the number and rate of deaths for all types of disasters such as drought, flood, storm, and hurricane; Borden and Cutter [18] analyzed the spatial trends in death rates due to disasters in the US, and another study [9] analyzed various disasters in the US over 25 years, evaluating gender, race, and age characteristics, as well as vulnerable areas. However, these researchers did not assess the specific causes of death according to diverse types of disasters.

It is also necessary to examine changing trends in the numbers and characteristics of deaths due to meteorological disasters by analyzing long-term data, in order to assess the influences of climate change on health, predict changes in future damages, and set up appropriate countermeasures.

The present study analyzed specific causes, and demographic and regional characteristics of death due to meteorological disasters in South Korea from 1990 to 2008, compared those characteristics according to the types of meteorological disasters, and analyzed changing trends with regards to deaths.

## Methods

### Data collection

In Korea, the local governments dispatch their civil servants to check accident scenes and prepare and submit Victim Survey Reports when people are killed in meteorological disasters. Civil servants identify death by a meteorological disaster based on the relatively clear causal relationship between the meteorological disaster and the accident or damage that directly caused the victim's death.

A Victim Survey Reports is prepared on the basis of the accident and contains information concerning the time of death, place of accident, name, gender, and address of the deceased, details and cause of the accident, photos of the accident scene, meteorological conditions at the time of the accident, and measures taken by the local government. A uniform report form is used across the nation. The 16 metropolitan governments combine those reports and submit them to the National Emergency Management Agency (NEMA). Based on these reports, NEMA has collected all information of the victims of meteorological disasters. In the present study, information from all deaths from 1990 to 2008 were re-processed and analyzed for our specific research goals. After excluding data from 2001 where some information was missing, a total of 2,045 deaths were examined for cause, location, and year of accident, as well as gender and age of victims. Deaths also included subjects who were missing for a long period of time.

### Categories of Meteorological Disasters

The types of meteorological disasters, which could be the cause of death, were selected based on official annual meteorological reports by the Korea Meteorological Administration at the time of death. Due to the high level of complexity and ambiguity of the categories proposed by administration investigators, similar type disasters were combined, placing them in the same category in order to facilitate comparison of results with international studies. Meteorological disasters such as strong wind, storms, and wind and waves meet the criterion of wind speed of 14 m/s on land or 21 m/s in the sea, and thus they were combined under the category of storm. Heavy rain defined by the Korea Meteorological Administration as precipitation of 80 mm for 12 hours or 150 mm, is referred to as flood in this study. Meteorological disasters caused by winter cold such as heavy snow, snowstorms, and cold are grouped together as cold. Since a typhoon is a phenomenon of strong wind, wind and waves, and heavy rain occurring together, those meteorological events were placed in the typhoon category (Table 1). These categories are similar to those used by Thacker et al. [9] for defining the categories of storm and flood, and cold and lightning, with the exception that, unlike their study, our study distinguishes storm from flood.

**Table 1:** Categories of Meteorological Disasters

Categories used in the present study	Categories used by the Korea Meteorological Administration
storm	strong wind, wind and waves, storm

cold	heavy snow, snowstorms, cold

flood	heavy rain

typhoon	typhoon

lightning	lightning

### Causes of Death

Causes of death listed on victim survey reports do not follow the systematic categories used in medicine, but adopt diverse expressions to depict the varied situations surrounding the deaths. Therefore, based on the categories described in previous studies [9,16] and the Disaster-Related Mortality Surveillance [19] by the Centers for Disease Control and Prevention of the U. S. A., the present study categorized specific causes of death into drowning, structural collapse, electrocution, lightning, fall, collision, landslide, avalanche, deterioration of disease by disaster, and others. Drowning by place of occurrence was further categorized into drowning in a river, sea, submerged house, sunken vessel, or urban facility such as manholes, roads, or sewer. When deaths occurred as a result of meteorological disasters resulting in the collapse of a bridge, wall, bungalow, stable, terrace, bank, house, or flooding causing collapse of a house and/or road, these deaths were all combined in the category of structural collapse. When death occurred due to being hit by an object, having limbs severed, or being hit by a vehicle, these deaths were combined in the category of collision. Electrocution, lightning, fall, avalanche, deterioration of disease by disaster, and landslide were analyzed as separate causes of death, since the cause of death in such cases was clear (Table 2).

**Table 2:** Causes of death by meteorological disasters

Causes of death	Examples of expressions for causes of death on victim survey reports
Drowning in a river	Caught in a swift stream while crossing a river on foot or on a bike, caught in a swift stream on a submerged cultivator, caught in a flooded river or swift stream while camping, driving into a river while victims are operating a motor vehicle, crossing a submerged bridge, caught in a swift stream while working on a bridge, missing and/or dying in a swift stream while working on a rice field, and dying in a swift stream while on patrol of a river

Drowning in the sea	Missing in waves during fishing or tidal waves, missing and/or dying in waves while watching on the shore

Drowning in a sunken vessel	Sinking of a ship while working or while stranded, missing while escaping from the ship overturned by waves, communication interruption and missing at anchor, colliding with a breakwater being pulled by a tugboat, and sinking in high waves

Drowning in a submerged house	Death from shock in a flooded house and death in a flooded house due to river overflowing

Drowning in an urban facility	Losing one's footing and falling into a sewage manhole to death in a flood, drowning in sewage being swept by strong winds, overtaken by a swift stream while driving an automobile, drowning in a capsized vehicle on a sunken road, missing while removing foreign matter from a manhole on a construction site, and drowning while checking the floodgate at the water supply

Structural collapse	Collapse of bridge, wall, bungalow, flooded road, stable, stone embankment, bank, slope, house, and missing house and/or road

Electrocution	Electrocuted while moving fallen electric poles, electrocuted while doing repair on a roof top, and electrocuted while walking

Lightning	Hit by lightning while fishing on a breakwater, in a vinyl house, while harvesting rice, on a field, on a ship, and on a golf course

Fall	Death from a fall

Collision	Death from a shock caused by moving obstacles and death from the shock of a flying object

Avalanche	Factory collapse due to heavy snow and frozen to death following exhaustion while climbing out of an avalanche

Deterioration of disease by disaster	Death from heart-lung failure

Landslide	Death from the fall of an earth pile, buried in a house in a landslide, and buried in a landslide while camping

Other	Other causes of death

### Statistical Analysis

The specific causes of death were compared and analyzed according to types of meteorological disaster. Demographic and regional characteristics were also analyzed to examine relative vulnerability. Victims 0-4 years of age were placed in the young childhood age group and those 5-19 years of age in the adolescence age group. Groups were formed in 10-year increments for those victims ranging from 20-80 years of age. Regions were analyzed according to their geographic distribution based on 232 basic administrative units, including cities, *guns*, and *gus*, through the geographic information system(GIS). Regions were also divided into metropolitan cities, provinces (small- and medium-sized cities and farming and fishing towns), inland areas, and coastal regions. In order to calculate death rates per 1,000,000 individuals, the resident registration data for inhabitants over 5 years old of administrative district such as *dongs, eups*, and *myeons *in 2000 of the National Statistics Office were used, along with resident registration data of administrative district such as cities, *guns*, and *gus*. The annual average of deaths was compared and analyzed according to types of meteorological disasters between the 1990s and 2000s in order to determine if there were changes in the types of meteorological disasters that produced the most deaths. Statistical analysis was carried out with the SPSS 12.0 program and Excel.

## Results

Table 3 shows the causes of all deaths according to the types of meteorological disasters analyzed in the study. Deaths from flood were the greatest at 966 (47.2%), followed by typhoon at 748 (36.6%) and storm at 316 (15.5%). Drowning at 1,234 (60.3%) was the number 1 cause of death among deaths by meteorological disasters, followed by landslide at 403 (19.7%) and structural collapse at 206 (10.1%). The percentages of deaths by landslide (26.0%) and structural collapse (14.4%) were higher in floods than other types of meteorological disasters, and 96.6% of deaths in storms were due to drowning.

**Table 3:** Causes of death according to the type of meteorological disaster, 1990-2008

Causes of death	Flood	Typhoon	Storm	Cold	Lightning	Sum
Drowning	505 (52.2)	424 (56.7)	305 (96.6)	0	0	1,234 (60.3)

in a river	478 (94.7)	263 (62.0)	2 (0.7)	0	0	743 (60.1)

in the sea	2 (0.4)	39 (9.2)	19 (6.2)	0	0	60 (4.9)

in a sunken vessel	15 (2.9)	69 (16.3)	284 (93.1)	0	0	368 (29.8)

in a submerged house	5 (1.0)	45 (10.6)	0	0	0	50 (4.1)

in an urban facility	5 (1.0)	8 (1.9)	0	0	0	13 (1.1)

Sub total	505 (100)	424 (100)	305 (100)	0	0	1,234 (100)

Structural collapse	139 (14.4)	64 (8.6)	2 (0.6)	1 (12.5)	0	206 (10.1)

Electrocution	23 (2.4)	18 (2.4)	0	0	0	41 (2.0)

Lightning	17 (1.8)	4 (0.5)	1 (0.3)	0	7 (100)	29 (1.4)

Fall	2 (0.2)	2 (0.3)	0	0	0	4 (0.2)

Collision	1 (0.1)	10 (1.3)	0	0	0	11 (0.5)

Landslide	251 (26.0)	149 (19.9)	3 (0.9)	0	0	403 (19.7)

Avalanche	0	0	0	6 (75.0)	0	6 (0.3)

Deterioration of disease by disaster	0	0	1 (0.3)	0	0	1 (0.1)

Other	28 (2.9)	77 (10.3)	4 (1.3)	1 (12.5)	0	110 (5.4)

Sum	966 (100)	748 (100)	316 (100)	8 (100)	7 (100)	2,045 (100)

Drowning was found to be the most important cause of death by meteorological disaster and was further analyzed according to location. Of the 1,234 people that drowned, those who drowned in a river and in a sunken vessel totaled 743 (60.1%) and 368 (29.8%), respectively. Of the 505 deaths by drowning in a flood, 478 (94.7%) occurred in a river. Of the 424 deaths by typhoon, 263 (62.0%) occurred in a river, with the remaining individuals drowning in a sunken vessel or submerged house. Most deaths by storm were due to drowning, and 93.1% occurred in a sunken vessel. As for gender, men accounted for 60.8% of deaths by "drowning in a river," whereas women accounted for only 38.9%. Men accounted for 65.5%, 96.5%, and 54.1% of deaths by "drowning in the sea," "drowning in a sunken vessel," and "landslide," respectively, with greater percentages for each category compared with women. Women accounted for 52.4% and 56.0% of deaths by "structural collapse" and "drowning in a submerged house," respectively, percentages that were slightly greater than those for men. These findings show that in women, death occurs more frequently in residential areas, whereas in men, death occurs more frequently outdoors.

Table 4 presents the results of the analysis of deaths by meteorological disaster according to demographic characteristics. While 3.11 per 1 million men died due to a meteorological disaster, the number was 1.63 for women, making the male death rate 1.9 times higher than the female death rate. The death rate increased with age; specifically, there were 1.71 deaths per 1 million individuals in their twenties, 2.71 in their forties, 3.68 in their fifties, and 4.70 in their sixties. A similar tendency was seen with flood and typhoon disasters. The number of males who died by storm was significantly higher than that for females, with the highest number of deaths in the forties and fifties age groups.

**Table 4:** Death rate * according to the type of meteorological disaster by gender and age, 1990-2008

	Total population	Flood	Typhoon	Storm	Cold	Lightning	Sum
**Gender**							

Male	23,962,088	1.28	1.12	0.69	0.01	0.01	3.11

Female	23,770,470	0.96	0.61	0.04	0.01	0.01	1.63

**Age**							

00-04	3,228,008	0.67	0.39	0.12	0.00	0.00	1.18

05-19	10,462,952	0.91	0.03	0.02	0.01	0.00	0.97

20-29	8,366,096	0.79	0.45	0.45	0.02	0.00	1.71

30-39	8,875,139	0.83	0.67	0.53	0.00	0.00	2.03

40-49	7,185,831	1.13	0.87	0.67	0.01	0.03	2.71

50-59	4,447,020	1.49	1.54	0.61	0.00	0.04	3.68

60-69	3,157,953	2.36	2.16	0.18	0.00	0.00	4.70

70-79	1,502,263	2.22	2.63	0.22	0.00	0.00	5.07

80+	507,296	4.38	5.14	0.22	0.00	0.00	9.74

* Annual average deaths per 1 million people calculated using the 2000 resident registration data for individuals over 5 years old of the Korean Statistical Information Service.

**Figure 1:**
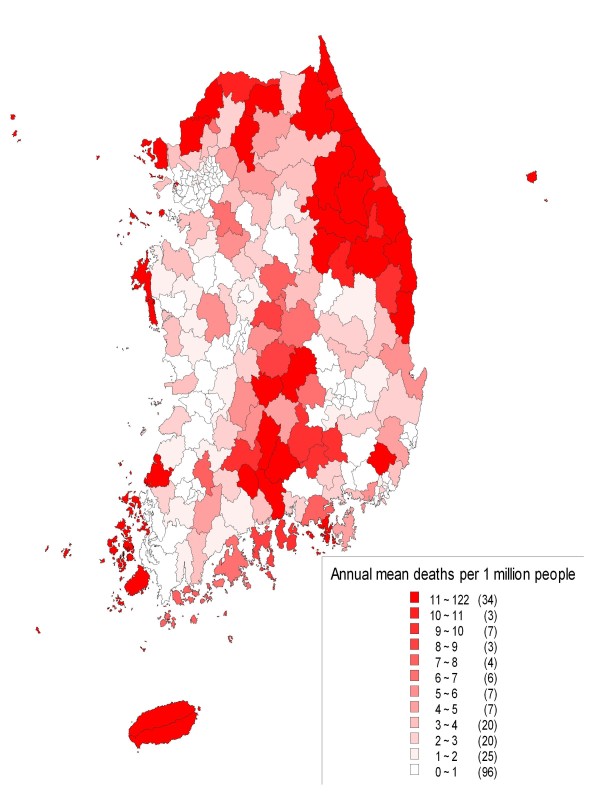
Death rates* in administrative units of cities, *guns*, and *gus*, 1990-2008. * Annual mean deaths per 1 million people calculated using the 2000 resident registration data of the Korean Statistical Information Service.

Figure 1 shows the geographic distribution of death rates by meteorological disasters according to the basic administrative units of Korea including cities, *guns*, and *gus*.

Table 5 shows the comparison of death rates between metropolitan cities, provinces (small- and medium-sized cities and farming and fishing towns), inland regions, and coastal regions of the 232 basic administrative units. The coastal regions of provinces (small- and medium-sized cities and agricultural and fishing towns) recorded the highest death rate due to disasters at 6.19 per 1 million people, followed by the inland regions of provinces at 3.23, the coastal regions of metropolitan cities at 0.94, and the inland regions of metropolitan cities at 0.55. Death rates due to meteorological disasters were higher in provinces than metropolitan cities and in coastal regions compared to inland regions. The overall death rate showed the same tendency as that for typhoons, which had a 0.26 death rate in the inland regions of metropolitan cities, 0.39 in the coastal regions of metropolitan cities, 0.73 in the inland regions of provinces, and 3.11 in the coastal regions of provinces. In the case of storms, most deaths occurred in coastal regions, and the coastal regions of provinces accounted for the majority of deaths, at 2.17 per 1 million individuals. The death rate by flood was 0.35 and 0.29 in the coastal and inland regions of metropolitan cities, respectively, with the coastal regions recording a slightly higher death rate. In provinces, the death rate of inland regions was 2.47 per 1 million individuals, which was much higher than the 0.84 of the coastal regions.

**Table 5:** Death rates* of regions according to meteorological disaster types, 1990-2008

Region Classification		Total population	Flood	Typhoon	Storm	Cold	Lightning	Sum
Metropolitan city	inland	15,593,082	0.29	0.26	0.00	0.00	0.00	0.55
	
	coastal	7,382,500	0.35	0.39	0.20	0.00	0.00	0.94

Province	inland	16,374,713	2.47	0.73	0.01	0.00	0.02	3.23
	
	coastal	7,314,474	0.84	3.11	2.17	0.06	0.01	6.19

* Annual mean deaths per 1 million people calculated using the 2000 resident registration data of the Korean Statistical Information Service.

**Figure 2:**
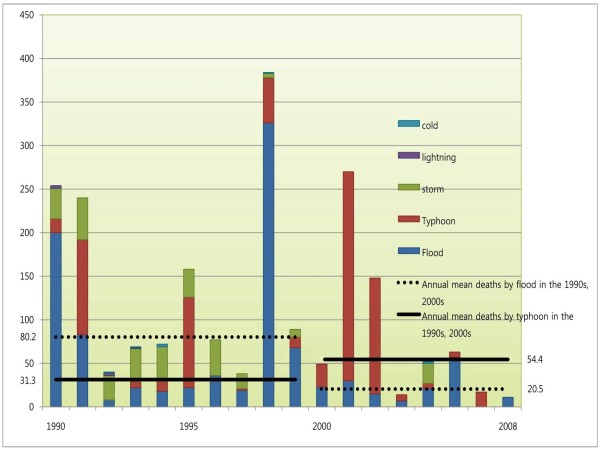
**Change in the number of annual average deaths due to meteorological disasters, 1990-2008**.

Figure 2 shows the changes in annual mean deaths by meteorological disasters. Deaths from all meteorological disasters were considerably decreased in the 2000s compared to the 1990s. Annual mean deaths by flood were 80.2 in the 1990s, decreasing to 20.5 by the quarter of the 2000s; and those by storm were 29.3 in the 1990s, decreasing by nine-tenths to 2.88 in the 2000s; but those by typhoon were 31.3 in the 1990s, increasing by 1.7 times to 54.4 in the 2000s.

## Discussion

The results of the present study indicate that, of all the meteorological disasters striking South Korea, floods caused the greatest number of deaths (966, 47.2%), with the majority due to drowning (505, 52.2%). These results are similar to previous studies that reported that two-thirds of all deaths from floods were due to drowning. In addition to floods, drowning was also the biggest cause of death for typhoons and storms, at 426 (56.7%) and 305 (96.6%), respectively. In the drowning category, "drowning in a river" accounted for 60.1%, which suggests a need to set up specific measures focused on avoiding river drowning when establishing preventive guidelines for dealing with meteorological disasters. Meanwhile, in the case of storms, 93.1% of those who drowned did so in a sunken vessel, which also emphasizes the need for preventive measures to address this cause of death.

A previous study in Australia [15] reported that men younger than 25 years of age and older than 59 years of age were vulnerable to flood damage. Another study in the US [9] reported that the male population 75 years of age or older was vulnerable to storms and floods. The fourth IPCC report also reported that the elderly were more vulnerable to damage and injury caused by meteorological disasters than younger age groups. On the other hand, a study on tsunamis [12] reported that women, infants, and young children were more vulnerable to this type of natural disaster. According to the results of the present study on the demographic vulnerability of victims of meteorological disasters, vulnerability was high among men and the elderly; particularly, in men, "drowning in a river" or "drowning in a sunken vessel," was more common compared to women, possibly because men tend to attempt more dangerous actions such as crossing an overflowing river. The fact that more men succumbed to "drowning in a sunken vessel" than women reflects the fact that in South Korea men typically work on fishing vessels that often sink during meteorological disasters. Since vulnerability differs according to the type of meteorological disaster, future preventive measures with regard to meteorological disasters should be based on vulnerability analysis results.

Regarding geographic vulnerability, provinces (small- and medium-sized cities and agricultural and fishing towns) and coastal regions were relatively more vulnerable to meteorological disasters than metropolitan cities and inland regions. These results correspond to predictions that climate change will most likely affect coastal regions [1,5,20-22].

The results of the analyses of the number and characteristics of deaths by meteorological disasters over the evaluated years confirm that the most deadly disaster has gradually moved from flood to typhoon. Annual mean deaths due to floods were decreased in the 2000s compared to the 1990s, because 37 floods occurred in the 1990s, a frequency that decreased by one-half to 18 in the 2000s; the mean deaths per occurrence were 21.7 in the 1990s, which decreased by more than half to 9.1 in the 2000s. On the contrary, annual mean deaths by typhoons increased, because the frequency of typhoons rose, with the mean deaths per occurrence 24.1 in the 1990s, which increased by 1.8 times to 43.5 in the 2000s. The present study also confirmed that the seasonal period of most deaths changed (data not shown). Annual mean deaths in August were 32.9 in the 1990s, which decreased to 11.0 in the 2000s, while they were 13.2 in September in the 1990s, which increased to 16.1 in the 2000s. The fact that the month with the most deaths moved from August (summer) to September (fall) is in line with the fact that more floods occur in the summer, whereas the fall is subject to more typhoons.

The data used in the present study have several strengths, in that the scenes where deaths occurred due to meteorological disasters were inspected first-hand. Our analysis included all deaths from accidents or injuries that had a relatively clear causal relationship with meteorological disasters in Korea. Previous studies gathered death data from different sources such as newspapers, articles, scientific and government reports, death certificates, or databases compiled from several national datasets. Thus, these researchers were not able to apply standardized death categories and therefore these studies had limitations that stemmed from inconsistent hazard mortality data. However, our study gathered data from the same database and utilized the total number of deaths, with the advantage of identifying causes of death in a consistent and specific manner.

Another advantage of the present study lies in the fact that we analyzed causes of death according to all types of meteorological disasters in detail, unlike previous studies [16,23-25]. Not only did we analyze the causes of death by separating the places of drowning, a major cause of death, but we also assessed the causes of landslide and structural collapse that accounted for approximately 40% of deaths other than drowning.

However, the present study is not without limitations. First, the analysis period was relatively short, from 1990 to 2008, compared to previous study [2], who analyzed data from 1900 to 2006, and another study in the US [9] that analyzed data from 1974 to 2004. nevertheless, the period analyzed in the current study appears to be long enough to identify causes of death and determine the demographic characteristics of these deaths, which was proven by the fact that changing trends in human casualties by meteorological disasters appeared in a clear and logical fashion according to decade.

Second, the study failed to include social and environmental elements of deaths by meteorological disasters. Vulnerability, such as the influence on health by meteorological disasters, depends on personal characteristics (location of residence, age, income, education, and disability) and social and environmental elements (level of preparation for disasters, responses for health, and environmental collapse) of individuals in danger [26-31]. Information concerning elements of individual behavior that leads to vulnerability, such as drinking and swimming skills, can be useful when developing behavioral guidelines for citizens. However, the present study failed to reflect those elements due to limitations of system data that did not consider disaster epidemiology. That limitation, however, applies to most of the previous studies on damage by meteorological disasters, as well as the present study. In the future, research on data system establishment and disaster epidemiology should be conducted to systematically collect personal characteristics and social and environmental information.

## Conclusions

The total number of deaths in Korea from meteorological disasters between 1990 and 2008 was 2,045. Floods caused the greatest number of deaths, but the greatest meteorological cause of death is slowly changing to typhoons. The most common cause of death was drowning. Factors associated with greater vulnerability were living in coastal provinces, older age, and male gender. A disaster epidemiology system is needed to establish effective measures for the adaptation to meteorological disasters.

## List of abbreviations

NEMA: National Emergency Management Agency; GIS: geographic information system.

## Competing interests

The authors declare that they have no competing interests.

## Authors' contributions

JYJ and HNM designed and coordinated the study. HNM analyzed and interpreted the data.

JYJ and HNM wrote the manuscript and the revision. Both authors approved the final manuscript.
